# *In silico* identification and verification of ferroptosis-related genes in type 2 diabetic islets

**DOI:** 10.3389/fendo.2022.946492

**Published:** 2022-08-05

**Authors:** Meiqi Yin, Liang Zhou, Yanan Ji, Rongxin Lu, Wei Ji, Guorong Jiang, Jin Ma, Xiudao Song

**Affiliations:** ^1^ Department of Endocrinology, Suzhou TCM Hospital Affiliated to Nanjing University of Chinese Medicine, Suzhou, China; ^2^ Clinical Pharmaceutical Laboratory of Traditional Chinese Medicine, Suzhou TCM Hospital Affiliated to Nanjing University of Chinese Medicine, Suzhou, China; ^3^ Department of Pharmacology, Nanjing University of Chinese Medicine, Nanjing, China; ^4^ Division of Rheumatology, The Affiliated Hospital of Nanjing University of Chinese Medicine, Nanjing, China; ^5^ Department of Pharmacy, Children’s Hospital of Soochow University, Suzhou, China

**Keywords:** type 2 diabetes, islet, ferroptosis, bioinformatical analysis, differential expression genes, hub gene

## Abstract

Type 2 diabetes (T2D) is a major global public health burden, with β-cell dysfunction a key component in its pathogenesis. However, the exact pathogenesis of β-cell dysfunction in T2D is yet to be fully elucidated. Ferroptosis, a recently discovered regulated form of non-apoptotic cell death, plays a vital role in the development of diabetes and its complications. The current study aimed to identify the key molecules involved in β-cell ferroptosis3 in patients with T2D using the mRNA expression profile data of GSE25724 by bioinformatic approaches. The differentially expressed mRNAs (DE-mRNAs) in human islets of patients with T2D were screened using the islet mRNA expression profiling data from the Gene Expression Omnibus and their intersection with ferroptosis genes was then obtained. Ferroptosis-related DE-mRNA functional and pathway enrichment analysis in T2D islet were performed. Using a protein-protein interaction (PPI) network constructed from the STRING database, Cytoscape software identified ferroptosis-related hub genes in the T2D islet with a Degree algorithm. We constructed a miRNA-hub gene network using the miRWalk database. We generated a rat model of T2D to assess the expression of hub genes. A total of 1,316 DE-mRNAs were identified in the islet of patients between T2D and non-T2D (NT2D), including 221 and 1,095 up- and down-regulated genes. Gene set enrichment analysis revealed that the ferroptosis-related gene set was significantly different in islets between T2D and NT2D at an overall level. A total of 33 ferroptosis-related DE-mRNAs were identified, most of which were significantly enriched in pathways including ferroptosis. The established PPI network with ferroptosis-related DE-mRNAs identified five hub genes (JUN, NFE2L2, ATG5, KRAS, and HSPA5), and the area under the ROC curve of these five hub genes was 0.929 in the Logistic regression model. We constructed a regulatory network of hub genes and miRNAs, and the results showed that suggesting that hsa-miR-6855-5p, hsa-miR-9985, and hsa-miR-584-5p could regulate most hub genes. In rat model of T2D, the protein expression levels of JUN and NFE2L2 in pancreatic tissues were upregulated and downregulated, respectively. These results contribute to further elucidation of ferroptosis-related molecular mechanisms in the pathogenesis of β-cell dysfunction of T2D.

## Introduction

Type 2 diabetes (T2D) has become a major public health burden developing into a worldwide epidemic and is now the leading cause of morbidity and mortality in the world. T2D affected 536.6 million people in 2021 and is projected to globally affect up to 783.2 million people by 2045 (1). Besides being increased risk of diabetic complications such as neuropathies and cardiovascular disease, people with T2D are also at an increased risk of developing some types of cancer, chronic hypertension, and non-alcoholic steatohepatitis (2, 3). Global expenditure on diabetes-related health problems in 2021 was approximately USD 966 billion and is projected to reach USD 1,054 billion by 2045 (1). Therefore, the discovery of the molecular mechanisms underlying the pathology of T2D may provide new therapeutic strategies for the treatments in this global health challenge. Although it is well established that the etiology of T2D is attributable mainly to progressive β-cell dysfunction, the exact pathogenesis of β-cell dysfunction in T2D remains to be fully elucidated. Therefore, the treatment of T2D would benefit from a comprehensive understanding of β-cell dysfunction at the molecular level.

In 2003, a new synthetic compound, erastin, was found to induce a form of non-apoptotic cell death that was first named ferroptosis in 2012 (4, 5). Ferroptosis is characterized by labile iron overload, accumulation of iron-dependent lipid peroxidation products, and a redox imbalance. Iron death is a research topic that has attracted much attention in recent years, and has been extensively studied in many pathogenic processes. Accumulating evidence has demonstrated an essential role of ferroptosis in the progression of diseases, such as cancer, and neurodegenerative and cardiovascular diseases, suggesting potential therapeutic strategies for targeting ferroptosis to prevent and treat these diseases. Emerging data suggest that iron death may play a vital role in the development of diabetes and its complications (6, 7). Interestingly, pancreatic islet β-cells often have decreased levels of antioxidant enzymes companied by the generation of ROS, suggesting that beta cells are more sensitive to ferroptosis. Furthermore, iron overload has been found in patients with T2D, particularly in pancreatic islets (8). In this regard, the ferroptosis inducer erastin was reported to induce ferroptosis in MIN6 β cells (9) and decrease glucose-stimulated insulin secretion in human islet, which was rescued by ferrostatin-1 (the ferroptosis inhibitor) (10), indicating that β-cell ferroptosis has a direct role of in β-cell dysfunction. Importantly, ferroptosis was observed in islets of a diabetic model and high glucose-induced β cell model (11, 12), highlighting that ferroptosis may be a promising target for T2D therapy. However, further advances in ferroptosis-targeting strategies for T2D require a detailed understanding of the expression patterns of ferroptosis-related genes expression in T2D islet. Exploration of the molecular mechanisms leading to β-cell dysfunction in T2D from the perspective of ferroptosis therefore has significant clinical potential.

So far, there are no bioinformatic studies on the ferroptosis-related genes in the islet of T2D. Therefore, we have used GSE25724 mRNA expression profiling data from the Gene Expression Omnibus (GEO) to identify ferroptosis-related genes between T2D islet and non-T2D (NT2D) islet samples. Gene Ontology (GO) annotation, Kyoto Encyclopedia of Genes and Genomes (KEGG) pathway enrichment analysis, and correlation analysis of the differentially expressed mRNAs (DE-mRNAs) related to ferroptosis in the islet of T2D were performed, and a protein-protein interaction (PPI) network was established. Then, we identified ferroptosis-related hub genes. Then microRNAs (miRNAs) and transcription factors (TFs) were predicted. We used a rat model of T2D to assess the expression levels of hub genes in islet. Overall, this study should help to understand the molecular level of ferroptosis in islet of T2D, contributing to the development of a theoretical basis for ongoing basic research on ferroptosis of T2D and ferroptosis-targeting T2D treatments.

## Materials and methods

### Microarray data source

We searched the GEO database using the following keywords “((Diabetes) AND Islet) AND “Homo sapiens”[porgn::txid9606]”, and “Expression profiling by array” to obtain the gene expression datasets of diabetic islet. After the systematic review, five GEO datasets (GSE118139, GSE38642, GSE41762, GSE50397 and GSE25724) associated with diabetic islet were found. As the low number of samples for islets in GSE118139, and low number of differentially expressed genes analyzed by GEO2R for GSE38642, GSE41762 and GSE50397, these four GSE profiles were excluded. The mRNA expression profiling data from the GSE profile (GSE25724) was selected and downloaded. GSE25724 was based on GPL96 [HG-U133A] Affymetrix Human Genome U133A Array. The array data for GSE25724 included 6 type 2 diabetic islets and 7 non-diabetic islets. GEO belongs to public databases. The patients involved in the database have obtained ethical approval. Our study was based on open-source data, so there are no ethical issues or other conflicts of interest. In addition, the principal component analysis (PCA) plot of 13 samples was performed using the “ggplot2” package (v3.3.3) in R version 3.6.3.

### Identification of differentially expressed mRNAs and ferroptosis-related genes

We used GEO2R, an web tool to identify the DE-mRNAs between T2D and non-diabetic islets. Data processing was performed as described previously (13). Genes with cut-off criteria of *P*-value <0.05 and |log fold change | > 1.0 were considered to be DE-mRNAs. DE-mRNAs volcano plots were constructed using a visual hierarchical cluster analysis

We obtained 259 ferroptosis-related genes from the Ferroptosis Database (FerrDb; http://www.zhounan.org/ferrdb/legacy/index.html) and intersected them with DE-mRNAs in GSE25724 to identify ferroptosis-related DE-mRNAs by a Venn diagram. Visual hierarchical cluster analysis showed the DE-mRNAs and ferroptosis-related DE-mRNAs in the volcano plot. A heat map of ferroptosis-related DE-mRNAs was generated by the “ComplexHeatmap” package in R version 3.6.3.

### Gene set enrichment analysis

Gene set enrichment analysis (GSEA) was used to analyze the gene expression data in GSE25724 using the C2 (CP) gene sets (C2. cp. v7.2 symbols. gmt) from the Molecular Signatures Database (MSigDB) of the cluster Profiler package (version 3.14.3). The items with the following criteria were regarded as significantly enriched: *P*-adjust value < 0.05 and FDR < 0.25.

### GO terms and KEGG pathway enrichment analysis

To obtain function and involved biological processes of ferroptosis-related DE-mRNAs, gene ontology (GO) annotation was performed using the “GO plot” (v1.0.2), “ggplot2” (v3.3.3) and “clusterProfile” (v3.14.3) packages in R version 3.6.3. The GO analysis included three categories: biological process (BP), cellular component (CC), and molecular function (MF). Kyoto Encyclopedia of Genes and Genomes (KEGG) was used for pathway enrichment analysis using the “GO plot” (v1.0.2), “org.Hs.eg.db” (v3.10.0) and “clusterProfile” (v3.14.3) packages in R version 3.6.3. The items with the following criteria were regarded as significantly enriched: *P*-adjusted value < 0.05.

### PPI network construction and hub genes identification

Using a Search Tool for the Retrieval of Interacting Genes (STRING), we obtained a functional protein association networks of ferroptosis-related DE-mRNAs. Ferroptosis-related DE-mRNAs were first uploaded to the STRING database, and then PPI pairs with a combined score greater than 0.4 were extracted. Then, using CytoHubba (a plug-in for Cytoscape v3.7.2), we identify hub genes by degree method (14).

### miRNA prediction of hub genes and miRNA-mRNA construction

The miRWalk 3.0 database (15) was used to predict miRNA of five hub genes using a score ≥ 0.95 as the cutoff criterion. The intersecting miRNAs were selected by the miRTarBase, TargetScan, or miRDB databases to ensure the accuracy and reliability of the results. Construction of a miRNA–hub gene regulatory network was performed using Cytoscape software.

### Molecular function, biological pathways, and prediction of potential transcription factors of miRNAs

The molecular functions, biological pathways, and upstream transcription factors of the miRNAs were predicted using FunRich software 3.1.3, which is mainly used for the analysis of functional enrichment and interaction network of genes and proteins (16).

### Animals

Adult male Sprague-Dawley rats (11 weeks old; weighing approximately ~200 g) were purchased from JOINN Laboratories, and were performed in accordance with the European Union Directive 2010/63/EU for animal experiments. All the animal experimental protocols were approved by The Local Committee on Ethics of Animal Experiments of Suzhou TCM Hospital Affiliated to Nanjing University of Chinese Medicine (Suzhou, China). The rats were allowed to eat and drink freely, and were fed with standard rat chow (Shuangshi Laboratory Animal Feed Science Co., Ltd.). After one week of acclimatization, rats were randomly assigned to the control group and T2D model group. Six rats in the T2D model group were fed by a high-fat diet (HFD, 60% fat) for 4 months, and then were intraperitoneally injected with 50 mg/kg streptozotocin (STZ); six rats in the control group were fed a standard rat diet and received physiological saline solution. The glucose level measured using a basic glucose level monitor after modeling is above 16.7 mM, confirming that the T2D model was successfully established.

### Western blot analysis

The pancreatic tissues of rats were collected, washed, and homogenized in radioimmunoprecipitation assay lysis buffer. Methods for quantification of whole protein content and western blot have been described previously (17). The primary antibodies used for western blotting were as follows: Anti-JUN (ab31419, Abcam), anti-NFE2L2 (ab92946, Abcam), anti-ATG5 (D5F5U, Cell Signaling Technology), anti-KRAS (#F234-sc30, Santa Cruz Biotechnology), and anti-HSPA5 (ab21685, Abcam). The intensity of the bands was analyzed using the SpotDenso tool of the built -in software of the detection instrument (Alphaimager 2200, ProteinSimple).

### Statistical analysis

The expression levels of ferroptosis-related DE-mRNAs between T2D and NT2D islets were compared using the Wilcoxon rank sum test. Correlation analysis of genes were carried out using Spearman’s method, and visualizations were generated using the package ggplot2 (v 3.3.3) package in R. The protein expression levels of hub genes were compared using unpaired t-test based on Prism (version 9.0; GraphPad Software, Inc.). *P* value < 0.05 was considered statistically significant.

## Results

### Identification of DE-mRNAs

**Figure 1:**
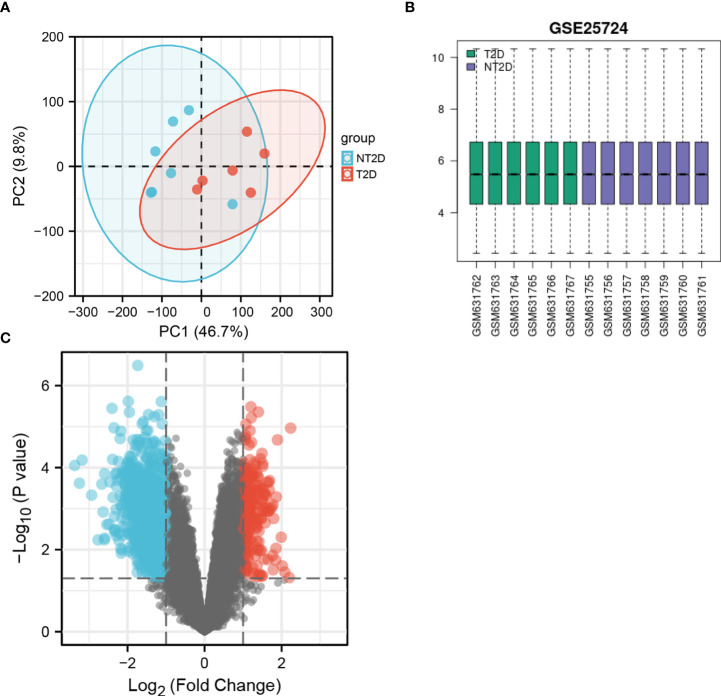
The PCA analysis and value distribution of the selected samples, and the volcano plot of the identified DE-mRNAs in GSE25724. **(A)** The visualization of principal component analysis for GSE25724. **(B)** The blue boxes represent NT2D samples and the pink boxes represent T2D samples. Black lines show the median of each data and its distribution represents the standardization degree of the data. **(C)** Volcano plot of the identification of DE-mRNAs, including 221 upregulated and 1,095 downregulated genes. Red, upregulation; green, downregulation.

**Figure 2:**
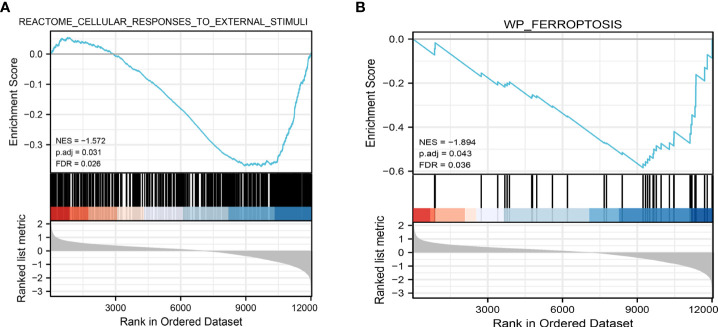
Representative results of GSEA analysis in the gene expression data in GSE25724. **(A)** The most significant enriched gene set negatively correlated with the T2D group was cellular responses to external stimuli (NES = -1.572, p.adj=0.031, FDR =0.026). **(B)** The ferroptosis-related gene set was negatively correlated with the T2D group (NES = -1.894, p.adj=0.041, FDR =0.036). NES, normalized enrichment score.

We performed PCA to evaluate the intra-group data repeatability, with the results showed that the repeatability of data in GSE25724 was satisfactory (**Figure 1A**). As shown in **Figure 1B**, the black lines are roughly on the same straight line, indicating that the standardization level was also satisfactory. The 12,383 genes representing the up- and down-regulation of genes in the GSE25724 dataset were marked in the red and blue, respectively (**Figure 1C**). After the GSE25724 dataset analyses, a total of 1,316 DE-mRNAs were identified, including 221 and 1,095 up- and down-regulated genes, respectively, as visualized in **Figure 1C** (**Supplementary Table 1**). The gene expression data in GSE25724 were analyzed holistically by GSEA using the C2 (CP) gene sets (MSigDB). As shown in **Figure 2A**, the most enriched item identified by GSEA was cellular responses to external stimuli (FDR =0.026), and complete GSEA results are provided in **Supplementary Table 2**. Based on GSEA analysis, the ferroptosis-related gene set was also enriched significantly at a nominal *P*-value <0.05 (FDR =0.036) in the T2D islet, and mostly downregulated (**Figure 2B**).

### Ferroptosis-related DE-mRNAs

**Figure 3:**
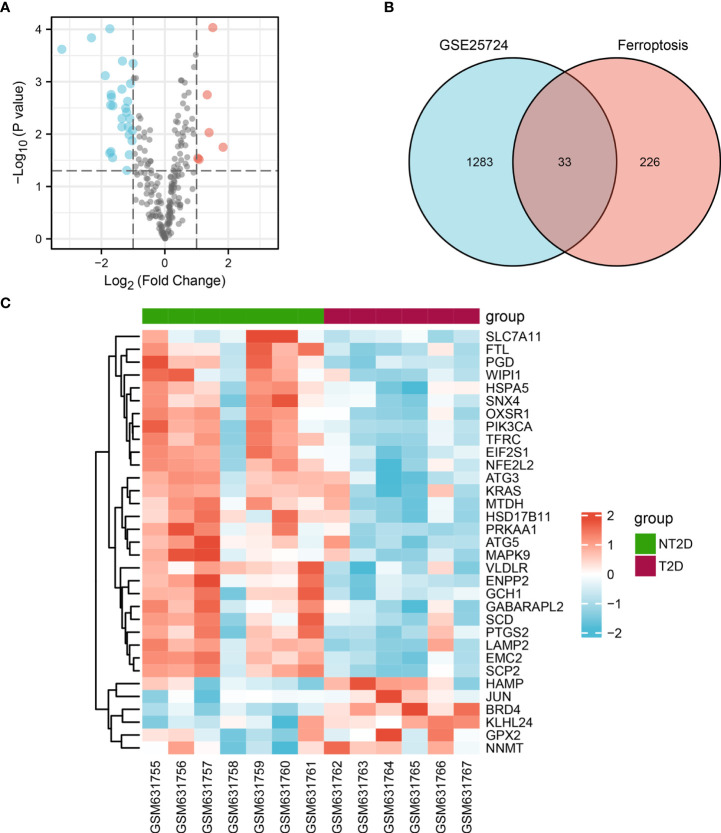
Ferroptosis-related DE-mRNAs between T2D islet and NT2D islet samples. **(A)** Volcano plot of 216 ferroptosis-related genes including six upregulated and 27 downregulated genes. Red, upregulation; green, downregulation. **(B)** Venn diagram to screen ferroptosis-related DE-mRNAs. **(C)** The heat map of 33 ferroptosis-related DE-mRNAs between T2D islet and NT2D islet samples.

**Figure 4:**
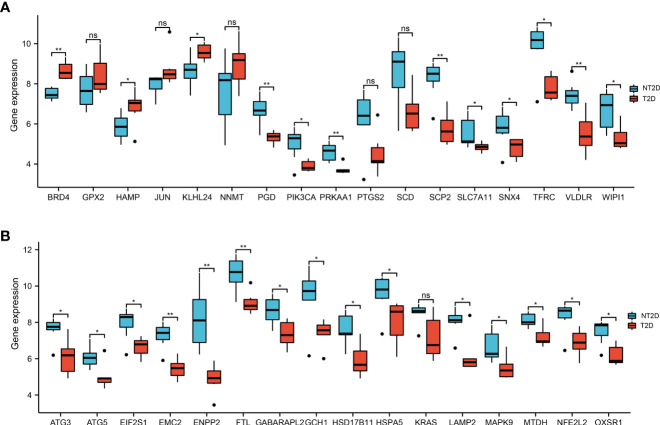
The boxplot of 33 ferroptosis-related DE-mRNAs in T2D and NT2D islet samples. **(A)** The boxplot of top 17 ferroptosis-related DE-mRNAs in T2D and NT2D islet samples. **(B)** The boxplot of last 16 ferroptosis-related DE-mRNAs in T2D and NT2D islet samples. Statistical analysis was performed with the Wilcoxon rank sum test. ns, not significant; ^*^ *P* < 0.05; ^**^ *P* < 0.01.

216 ferroptosis-related genes were found in GSE25724 and shown in Volcano Plot (**Figure 3A**). Next, we analyzed the expression of 216 ferroptosis-related genes in T2D and NT2D. After combining the analysis of the DE-mRNAs and 216 ferroptosis-related genes, we further identified 33 ferroptosis-related DE-mRNAs, including six that were upregulated and 27 that were downregulated (**Figure 3B**). The heat map of the 33 ferroptosis-related DE-mRNAs were shown in **Figure 3C**. Moreover, box plots showed differences in the expression patterns of 33 ferroptosis-related DE-mRNAs between the T2D and NT2D samples (**Figures 4A, B**). The 33 ferroptosis-related DE-mRNAs were classified further by the FerrDb online tool as either a ferroptosis driver, ferroptosis suppressor, or ferroptosis marker (**Table 1**).

**Table 1:** The ferroptosis-related DE-mRNAs were divided into ferroptosis driver, suppressor, and marker.

Marker	Driver	Suppressor
GPX2, HAMP, KLHL24, NNMT, EIF2S1, FTL, TFRC HSD17B11, OXSR1, PTGS2, VLDLR, NFE2L2, SLC7A11	ATG3, ATG5, EMC2, GABARAPL2, KRAS, MAPK9, MTDH, PGD, PIK3CA, PRKAA1, SCP2, SNX4, TFRC, WIPI1	BRD4, JUN, ENPP2, GCH1, HSPA5, LAMP2, NFE2L2, SCD, SLC7A11

### GO terms and KEGG pathway enrichment analysis of ferroptosis-related DE-mRNAs

**Figure 5:**
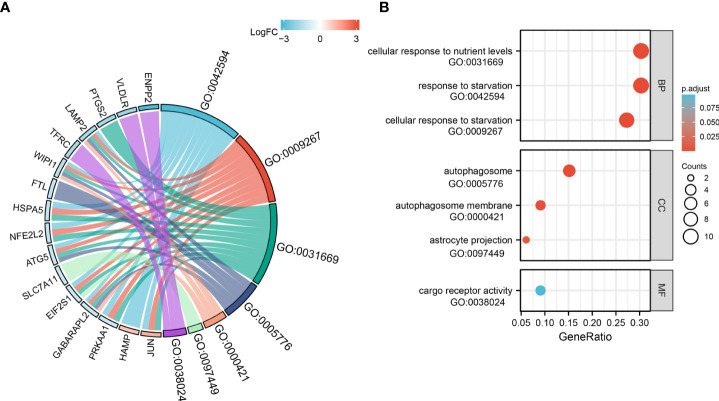
Gene Ontology (GO) functional analysis of 33 ferroptosis-related DE-mRNAs. Chord **(A)** and Bubble plot **(B)** of enriched GO terms of 33 ferroptosis-related DE-mRNAs. Y-axis: name of GO items; X-axis: percentage of the number of genes assigned to a term among the total number of genes annotated in the network; Bubble size, number of genes assigned to a pathway; Color: enriched -log10(*P*-value); Red bubble: indicates a greater significance level.

**Figure 6:**
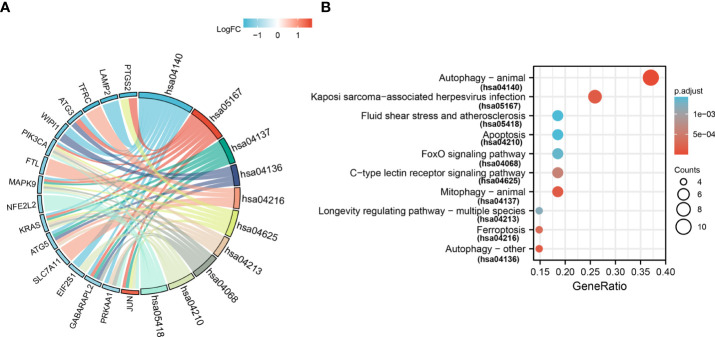
Kyoto Encyclopedia of Genes and Genomes analysis (KEGG) of 33 ferroptosis-related DE-mRNAs. Chord **(A)** and Bubble plot **(B)** of enriched KEGG terms of 33 ferroptosis-related DE-mRNAs. Y-axis: name of the KEGG signaling pathway; X-axis: percentage of the number of genes assigned to a term among the total number of genes annotated in the network; Bubble size, number of genes assigned to a pathway; Color: enriched -log10(*P*-value); Red bubble: indicates a greater significance level.

For GO-BP, the analysis revealed that these 33 ferroptosis-related DE-mRNAs were enriched significantly in the various biological process including cellular response to starvation, response to starvation, and cellular response to nutrient levels (**Figure 5**). In the GO CC analysis, the top three significantly enriched terms were autophagosome, autophagosome membrane, and astrocyte projection (**Figure 5**). No significantly enriched terms were identified in the GO MF analysis. The enriched KEGG items including ferroptosis, autophagy–animal, Kaposi sarcoma-associated herpesvirus infection, mitophagy–animal, and autophagy–other are shown in **Figure 6**.

### Correlation analysis, PPI network construction, and hub genes identification

**Figure 7:**
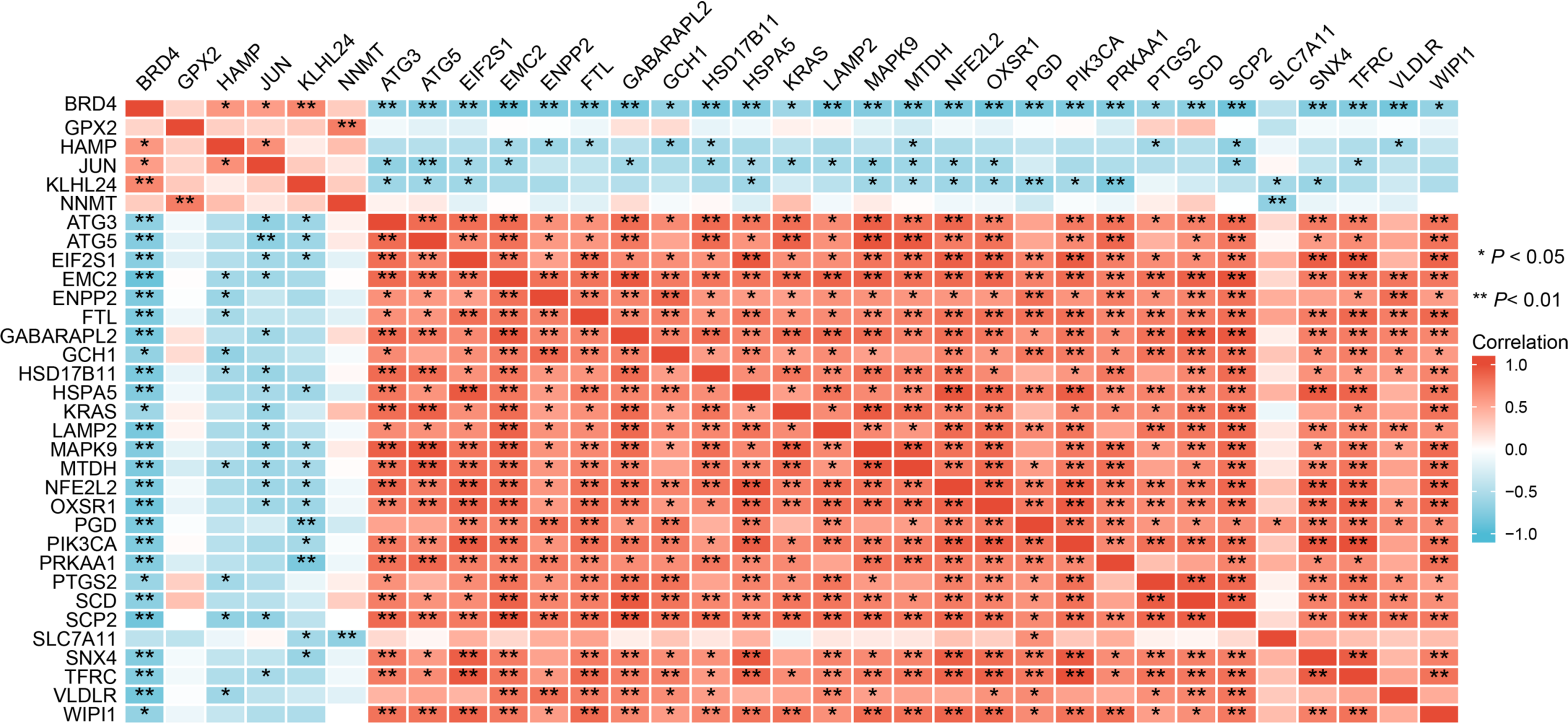
Spearman correlation analysis of the 33 ferroptosis-related DE-mRNAs.

**Figure 8:**
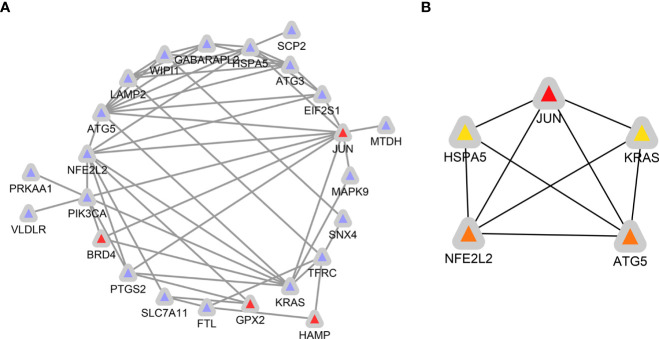
Identification of ferroptosis-related hub genes in diabetic islets. **(A)** PPI network constructed with ferroptosis-related DE-mRNAs were performed using the STRING. The nodes represent proteins, and the edges represent the interaction of the proteins. Blue, downregulation. red, upregulation. **(B)** Cytohubba in Cytoscape was used to find the top five hub genes in the PPI network by Degree. PPI network of the top 5 hub genes was visualized by Cytoscape, and the top 5 hub genes are displayed from red (high Degree value) to yellow (low Degree value).

The expression correlation of these 33 ferroptosis-related DE-mRNAs was investigated by a correlation analysis. The results showed the relationship between the 33 ferroptosis-related DE-mRNAs in the GSE25724 dataset (**Figure 7**), suggesting these 33 ferroptosis-related DE-mRNAs had synergistic interaction on expression. Using Cytoscape software, we have visualized the PPI network of DE-mRNAs obtained from the STRING database (**Figure 8A**). The PPI network contained 33 nodes (genes) and 55 edges (interactions), with a enrichment *P* value less than 2.08E-13. Using the Cytohubba plugin of Cytoscape, the top five nodes in the PPI network were generated by the degree method. JUN (AP-1 transcription factor subunit) had the highest connectivity degree (10), followed by (erythroid-derived)-like 2 (NFE2L2; 9), autophagy-related 5 (ATG5; 9), KRAS proto-oncogene (KRAS; 8), and Heat shock protein family A [Hsp70] member 5 (HSPA5; 8) (**Figure 8B**).

### Construction of the logistic regression for hub genes

**Figure 9:**
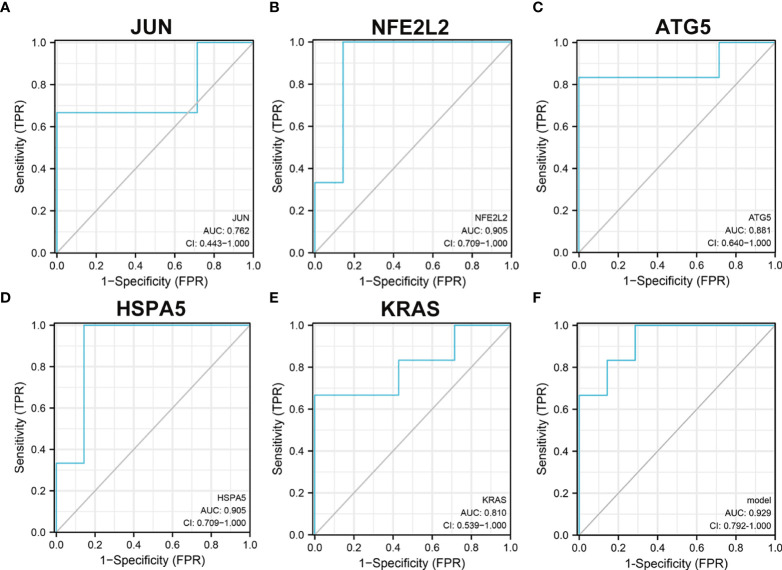
Receiver operating characteristic analysis revealed the predictive performance of ferroptosis-related hub genes for T2D. **(A)** The AUC of JUN **(A)**, NFE2L2 **(B)**, ATG5 **(C)**, KRAS **(D)**, and HSPA5 **(E)** in ROC monofactor analysis. **(F)** The AUC of five ferroptosis-related hub genes in the logistic regression model. Receiver operating characteristic, ROC; AUC, area under the ROC curve.

In univariate analysis of receiver operating characteristic (ROC), the areas under the ROC curves for JUN, NFE2L2, ATG5, KRAS, and HSPA5 were 0.762 (CI: 0.443−1.000), 0.905 (CI: 0.709−1.000), 0.881 (CI: 0.640−1.000), 0.905 (CI: 0.709−1.000), and 0.81 (CI: 0.539−1.000), respectively (**Figures 9A–E**). As shown in **Figure 9F**, these five hub genes were further selected to construct the logistic regression model. The area under the ROC curve of the model was 0.929 (CI: 0.792−1.000), which further indicates the predictive performance of five ferroptosis-related hub genes in T2D.

### Further miRNAs prediction of hub genes

**Figure 10:**
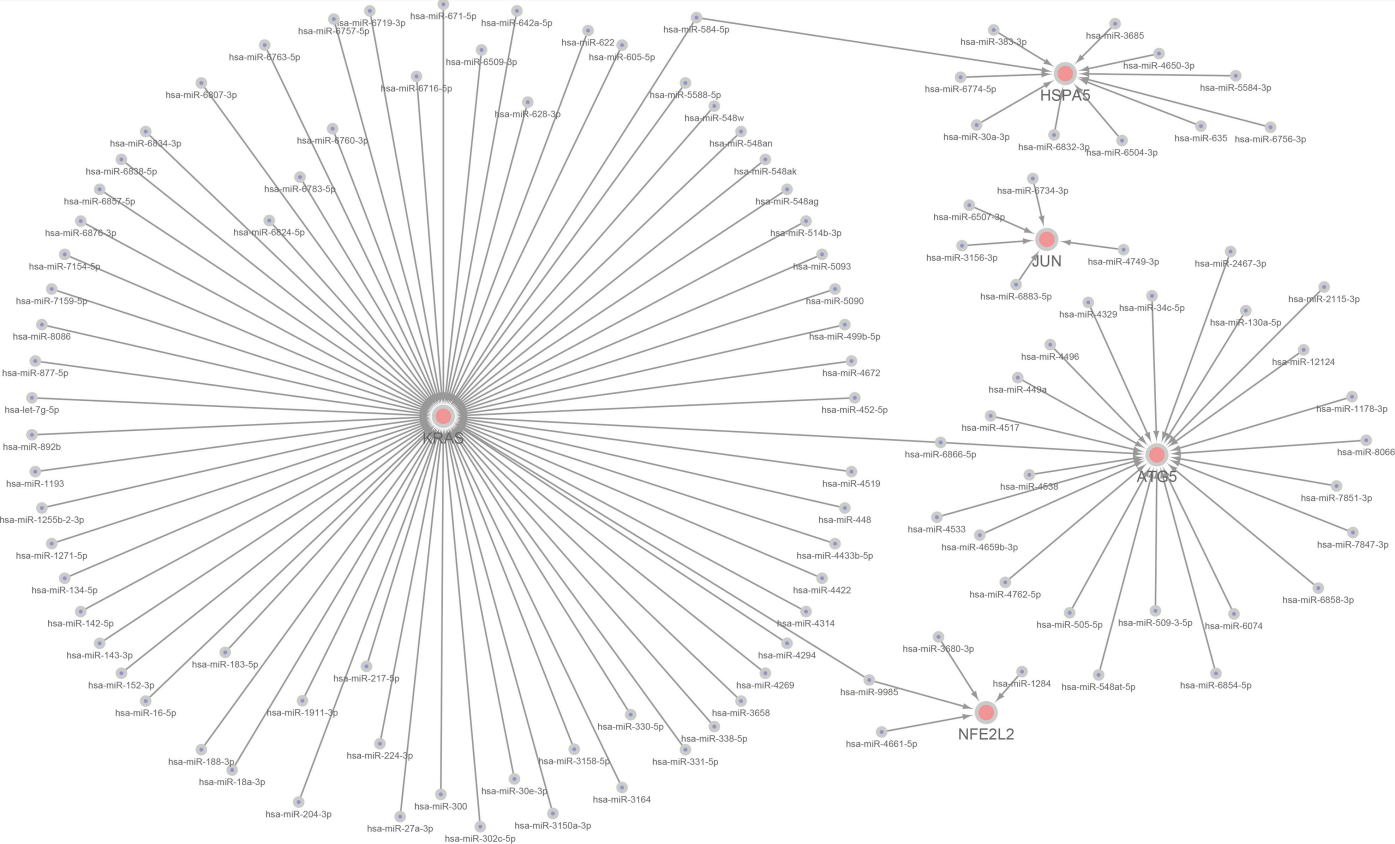
A miRNA–hub gene regulatory network construction in diabetic islets. Interaction network between hub genes and its targeted miRNAs. Genes were colored in red, miRNAs were colored in blue. Three higher amounts of cross-linked genes including hsa-miR-6855-5p, hsa-miR-9985, and hsa-miR-584-5p.

**Figure 11:**
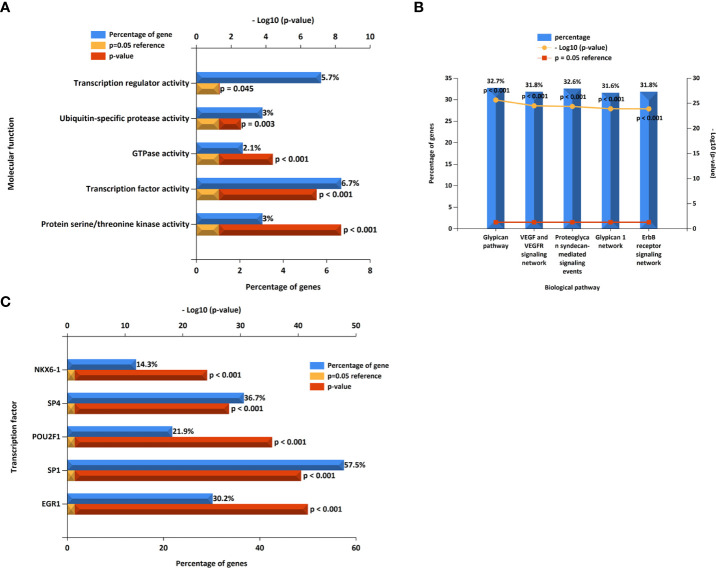
The molecular function, **(A)** biological pathways, **(B)** and prediction of potential transcription factors **(C)** of miRNAs.

We constructed a miRNA–hub gene regulatory network in T2D islets, and hsa-miR-6855-5p, hsa-miR-9985, and hsa-miR-584-5p had higher amounts of cross-linked genes (≥2) (**Figure 10**). Subsequently, we uploaded 113 miRNAs to Funrich, and the results of the enrichment analysis showed that the molecular functions were significantly enriched in protein serine/threonine kinase activity, transcription factor activity, GTPase activity, Ubiquitin-specific protease activity, and transcription regulator activity (**Figure 11A**). The biological pathways enriched included the Glypican pathway, VEGF and VEGFR signaling network, proteoglycan syndecan-mediated signaling events, Glypican 1 network, and ErbB receptor signaling network (**Figure 11B**). The top five transcription factors of these miRNAs included the EGR1, SP1, POU2F1, SP4, and NKX6-1, as shown in **Figure 11C**.

### Validation of hub genes

**Figure 12:**
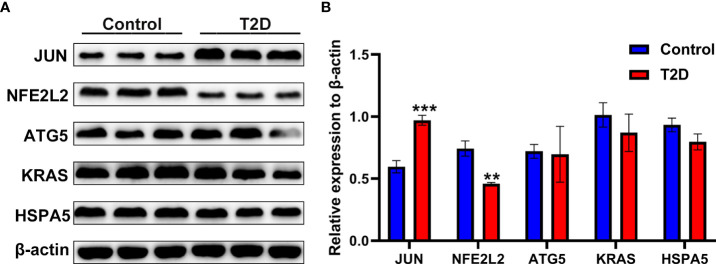
The protein expression levels of ferroptosis-related hub genes in a rat model of T2D. **(A)** The western blotting results of the protein expression levels of JUN, NFE2L2, ATG5, KRAS and HSPA5 in pancreatic tissues of a rat model of T2D generated by a high-fat diet and streptozotocin. **(B)** Semi-quantitative analysis of immunoblotting results of JUN, NFE2L2, ATG5, KRAS and HSPA5 in pancreatic tissues. ^**^ *P*< 0.01, ^***^ *P*< 0.001 vs. control group.

A rat model of T2D was used to validate five hub genes (JUN, NFE2L2, ATG5, KRAS, and HSPA5) at the protein expression level. As shown in **Figure 12**, the JUN protein level in the pancreatic tissue of the T2D model group was significantly higher than that of the control group. The NFE2L2 protein level in the pancreatic tissue of the T2D model group were significantly lower than that of the control group (**Figure 12**).

## Discussion

Ferroptosis is characterized by labile iron overload and accumulation of iron-dependent lipid peroxidation due to metabolic dysfunction. A previous study suggested a potential association between excessive body iron storage and an increased T2D risk (18). Recently, a mendelian randomization study further indicated there was a causal link between increased systemic iron status and enhanced risk of developing T2D (19). Clinical finding have shown an excessive iron deposition in T2D (8), and recent studies have suggested that ferroptosis might play a crucial role in the placental pathogenesis of β-cell dysfunction (11, 12). However, many ferroptosis genes have not yet been found, so further systematically analyzed ferroptosis in the islet of T2D is needed. The present study is the first to identify ferroptosis-related DE-mRNAs in the pancreatic islet of T2D using mRNA expression profile data of GSE25724. Consistently, genes associated with ferroptosis were enriched significantly when the RNA-seq data were analyzed with GSEA, which suggested an overall significant correlation between ferroptosis of islet and T2D. The present study further identified 33 DE-mRNAs involved in ferroptosis, including 6 upregulated and 27 downregulated genes. Furthermore, we identified five hub genes (JUN, NFE2L2, ATG5, KRAS, and HSPA5) from the constructed PPI network with these ferroptosis-related DE-mRNAs. Among five hub genes, the protein expression levels of JUN and NFE2L2 in pancreatic tissues of a rat model of T2D were consistent with the results of bioinformatics analysis of mRNA chip.

NFE2L2, (Erythroid-derived)-like 2, belonging to the cap-n-collar subfamily of transcription factors, was reported to be a crucial regulator protein of ferroptosis by regulating many vital genes related to iron/GSH homeostasis at the transcriptional level. The prevalence of NFE2L2 activating mutations across many solid tumors and its activation has also been linked to inhibition of the cancer cells growth by inhibiting ferroptosis (20). Several reports documented a ferroptosis-specific role of NFE2L2 in diabetic complications. Downregulation of NFE2L2 was observed in kidneys tissues of diabetic mice, with upregulation of NFE2L2 shown to inhibit ferroptosis and delay the progression of diabetic nephropathy (21). Zang et al. reported that activation of NFE2L2 resulting from autophagy, triggered ferroptosis in cardiomyocytes, causing cell death and myocardial damage and thereby worsening the progression of diabetic cardiomyopathy (22). Regarding pancreatic β cells dysfunction in diabetes, emerging evidence suggests that NFE2L2-mediated ferroptosis plays a crucial role in β-cell function. NFE2L2 protein expression was reported to be downregulated in islets of the diabetic model and a high glucose-induced INS-1 β cell model (12). Consistent with these findings, downregulated NFE2L2 expression was observed in islets of patients with T2D and pancreatic tissues of T2D rat model in the current study. Some substances have been found to protect β-cells function by inhibiting ferroptosis involving the NFE2L2 signaling pathway. The results of Yao et al. (23) showed the role of the NFE2L2 signaling pathway in protecting bilirubin (an endogenous heme metabolite) against β-cell ferroptosis, which may be a mechanism for the beneficial effect of bilirubin on diabetes). Zhou et al. reported that upregulation of the NFE2L2 signaling pathway by inhibiting ferroptosis was, at least in part, responsible for the protective effects of cryptochlorogenic acid on β-cells function in diabetes (12). The ferroptosis-specific role of NFE2L2 in diabetic islets is therefore worthy of further investigation.

Although the remaining four hub genes (JUN, ATG5, KRAS, and HSPA5) have been shown to affect ferroptosis pathway from studies performed on various diseases in the Ferroptosis Database, these genes have not been linked directly to the occurrence of ferroptosis in the diabetic islet. JUN (formally known as c-JUN), a subunit of the activator protein 1 transcription factor, has multiple roles in determining ferroptosis sensitivity through either transcription-dependent or transcription-independent mechanisms (24). It has been reported that overexpression of JUN inhibits erastin-induced ferroptosis in Schwann cells (25). JUN is also deduced as a ferroptosis suppressor in the Ferroptosis Database, which is based on the study performed on human liver cancer cells (26). Interestingly, JUN protein expression was increased in rat islet of T2D (27). Consistent with this finding, our study showed upregulated JUN expression in the islets of both rat models and patients with T2D. These results suggest a different role of JUN in mediating ferroptosis in disparate disease settings such as T2D. Accumulating evidence indicates that autophagy contributes to ferroptosis. Genetic depletion of the core autophagy effector molecules autophagy-related 5 (ATG5) in mouse embryonic fibroblasts resulted in inhibition of erastin-induced ferroptosis, iron accumulation, and lipid peroxidation, as well as subsequent ferroptosis (28). Clinical studies have shown that ATG5 expression was decreased in skeletal muscle from type 2 diabetes (29). Similarly, our study have showed that *ATG5* mRNA expression was identified to be downregulated in diabetic islets, suggesting a potential role of ATG5 as a ferroptosis inhibitor in the diabetic islet. The KRAS mutation is related closely to ferroptosis in several cancer cells. In the Ferroptosis Database, KRAS is deduced as a ferroptosis driver, which is based on the conclusion that KRAS12V mutant protects RMS13 cells from ferroptotic cell death (30). However, KRAS-mutant pancreatic cancer cells are sensitive to erastin or RSL3-induced ferroptosis (31), suggesting that KRAS may be a ferroptosis suppressor in pancreatic cancer cells. The downregulated KRAS expression in the islets of patients with T2D at the current study also suggests a ferroptosis suppressive role of KRAS in diabetic islet. Heat shock protein family A [Hsp70] member 5 (HSPA5), an endoplasmic reticulum (ER)-sessile chaperone, has been documented as a ferroptosis suppressor in the Ferroptosis Database, which is in agreement with the result that HSPA5 negatively regulates ferroptosis in human pancreatic ductal adenocarcinoma cells (32). Although the results of HSPA5 expression levels in islets of T2D were inconsistent, HSPA5 overproduction partially protected MIN6 β cells from lipid-induced apoptosis (33, 34). In the current study, *HSPA5* mRNA expression were identified to be downregulated in diabetic islets, suggesting that decreased HSPA5 expression contributes to trigger the occurrence of ferroptosis in diabetic islet. Interestingly, although some genes (SCP2,WIPI1, PRKAA1 and EMC2) were shown to be ferroptosis driver in the Ferroptosis Database, these genes were identified to be downregulated in T2D at the current study. This comparison suggests that these genes should have a different function in disparate disease settings. In agreement with our study, hepatic SCP2 expression was reduced in diabetic rat (35), WIPI1 expression were decreased in skeletal muscle from type 2 diabetes (29), decreased PRKAA1/AMPK phosphorylation was observed in high glucose-induced HK-2 cells (36). In addition, although EMC2 was validated to a ferroptosis driver in the non small cell lung cancer cell, EMC2 was identified to be upregulated in esophageal adenocarcinoma (37) and breast cancer (38). Thus, these results suggest the different role of these genes in mediating ferroptosis in T2D, but this hypothesis needs further investigation.

The xCT transporter, an anti-porter system composed of light chain xCT (encoded by SLC7A11) and heavy chain 4F2, has a well-established role in maintaining intracellular glutathione (GSH) levels and protecting cells from oxidative-stress-induced cell death, such as ferroptosis (39). Downregulation of SLC7A11 expression results in a reduction in intracellular cysteine levels, resulting in the cells being incapable of defending against oxidative stress, thereby sensitizing them to ferroptosis. SLC7A11 is frequently overexpressed in cancers that confer the ferroptosis resistance phenotype (39). It has been confirmed that patients with T2DM and its complications are devoid of GSH (40), with evidence also establishing that the role of SLC7A11-mediated ferroptosis in diabetes and its complications. Li et al. reported that downregulation of SLC7A11 expression and decreased GSH levels were observed in kidney tissues of diabetic mice and high glucose-induced HK-2 cells, which was rescued by the ferroptosis inhibitor Fer-1 (21). Ferroptosis was also evident in the heart of diabetic cardiomyopathy associated with the downregulation of SLC7A11 signaling (41). Advanced glycation end-products, an important pathogenic factor in diabetic cardiomyopathy, were found to induce ferroptosis in engineered cardiac tissues by downregulating SLC7A11 levels (41). The expression of SLC7A11 in bone tissue was also decreased markedly in the diabetic rat model (42). Metformin, an antidiabetic drug has a protective effect against palmitic acid in vascular smooth muscle cells by inhibiting ferroptosis, which is related to the downregulation of SLC7A11 expression (43). These findings suggest that downregulation of SLC7A11 contributes to the development of diabetic complications *via* triggering ferroptosis. Regarding pancreatic β cells dysfunction in diabetes, only one study has suggested that upregulation of xCT (SLC7A11) expression was observed in the islet of T2D mice (11). In the current study, expression of *SLC7A11* mRNA was identified to be downregulated in diabetic islets, suggesting a potential role of SLC7A11 in ferroptosis of the diabetic islet. Interestingly, GPX2, a member of the GPXs family which catalyzes the oxidation of glutathione rather than GPX4 (a common inhibitory mediator for ferroptosis), was identified to be upregulated in diabetic islet. In fact, it has been reported that silencing GPX2 partially reverses erastin-induced ferroptosis in lung adenocarcinoma, indicating a potential role of GPX2 as a ferroptosis driver in lung adenocarcinoma (44). The results of our study suggest that GPX2 has a ferroptosis driver role in diabetic islet, but this theory needs further investigation.

It is well established that intracellular iron overload triggers ferroptosis through several signaling pathways. Regulation of iron homeostasis includes iron storage, uptake by ferritin and transferrin receptor (TFRC, also known as TFR1). Ferritin consists of a ferritin light chain (FTL) that acts as a sink for iron when cellular iron levels are high. It has been demonstrated that ferritinophagy results in the degradation of ferritin complexes to release free iron, thereby causing increased sensitivity to ferroptosis. FTL expression has been reported to be upregulated in the islets of T2D mice (11). Iron-overload induced by ammonium iron (III) citrate consistently upregulated FTL expression in INS-1 β cells (45). Interestingly, the current study showed that *FTL* mRNA expression was decreased in diabetic islet, suggesting increased levels of intracellular free iron. TFRC is an iron uptake receptor related to ferroptosis. In diabetic rats, renal (46), myocardium (47), and hepatic (48) expression levels of TFRC were reported to be increased. Increased TFRC expression was observed in kidney tissues of diabetic mice and high glucose-induced HK-2 cells, which was rescued by the ferroptosis inhibitor Fer-1 (21). Treatment with high glucose resulted in the downregulation of TFRC expression in endothelial cells (49). An early study suggested that iron uptake by pancreatic β cells is also regulated and mediated by TFRC (50). TFRC protein expression has been reported to be upregulated in islet of diabetic model and high glucose-induced INS-1 β cells model (12). Interestingly, iron-overload induced by ammonium iron (III) citrate downregulated TFRC protein expression in INS-1 β cells (45). In the current study, consistent downregulation of *TFRC* mRNA expression was identified in diabetic islet due to the excessive iron deposition in the diabetic islet. It is therefore considered that intracellular iron overload in diabetic islet may be due to decreased expression of FTL.

## Conclusions

In summary, we identified five hub genes (JUN, NFE2L2, ATG5, KRAS, and HSPA5) that are closely associated with ferroptosis in diabetic islets, which are thus potential ferroptosis-related biomarkers for T2D therapy. Among these five hub genes, JUN and NFE2L2 were further validated to be consistent with the expression trend in GSE25724, highlighting the role of these two hub genes in regulating the ferroptosis of diabetic islets. Importantly, SLC7A11, GPX2, and FTL are NFE2L2 target genes, which suggests that downregulated NFE2L2/SLC7A11, NFE2L2/GPX2, and NFE2L2/FTL1 pathways may play a key role in the ferroptosis of diabetic islet combining with our present study. Thus, the role of the NFE2L2 signaling pathway in ferroptosis of diabetic islets needs further investigation using *in vitro* and *in vivo* experimental including knock down and overexpression assay. Only one GEO dataset was chosen for the current study, and we understand that this represents one more limitation of the current study. Also, rat sample size in the current study were not quite enough.

## Data availability statement

The original contributions presented in the study are included in the article/**Supplementary Material**. Further inquiries can be directed to the corresponding authors.

## Ethics statement

The animal study was reviewed and approved by The Local Committee on Ethics of Animal Experiments of Suzhou TCM Hospital Affiliated to Nanjing University of Chinese Medicine.

## Author contributions

XS and JM conceived and designed the experiments. MY, LZ, YJ, RL, WJ, GJ, and JM acquired and analyzed the data. MY, JM, and XS drafted the manuscript. XS are responsible for the integrity of the work as a whole. All authors read and approved the final manuscript.

## Funding

This work was supported by the research funds from the National Natural Science Foundation of China (grant nos. 82004315; 82104722), Gusu Health Talents Program Training Project in Suzhou (GSWS2021048), Jiangsu Provincial Medical Youth Talent (grant no. QNRC2016259), and Suzhou Science and Technology Department (SKJY2021129).

## Acknowledgments

We thank EditSprings (https://www.editsprings.com/yuyan) for its linguistic assistance during the preparation of this manuscript. We acknowledge GEO database for providing their platforms and contributors for uploading their meaningful datasets.

## Conflict of interest

The authors declare that the research was conducted in the absence of any commercial or financial relationships that could be construed as a potential conflict of interest.

## Publisher’s note

All claims expressed in this article are solely those of the authors and do not necessarily represent those of their affiliated organizations, or those of the publisher, the editors and the reviewers. Any product that may be evaluated in this article, or claim that may be made by its manufacturer, is not guaranteed or endorsed by the publisher.

## Supplementary material

Supplementary Table 1 Significantly differentially expressed mRNA in GSE25724.

Supplementary Table 2 Gene Set Enrichment Analysis results for the gene expression data in GSE25724.

Supplementary Table 3 Predicted miRNA for five hub genes.
